# Being Prodromal: Current Criteria in the Context of Isolated REM Sleep Behavior Disorder

**DOI:** 10.1002/mdc3.70006

**Published:** 2025-02-22

**Authors:** Anja Ophey, Sinah Röttgen, Julia Reichrath, Elke Kalbe, Gereon R. Fink, Michael Sommerauer

**Affiliations:** ^1^ Medical Psychology | Neuropsychology and Gender Studies, Center for Neuropsychological Diagnostics and Intervention (CeNDI), Faculty of Medicine and University Hospital Cologne, University of Cologne Cologne Germany; ^2^ Cognitive Neuroscience, Institute for Neuroscience and Medicine, INM‐3, Research Centre Juelich Juelich Germany; ^3^ Department of Neurology Faculty of Medicine and University Hospital Cologne, University of Cologne Cologne Germany; ^4^ Center of Neurology, Department of Parkinson, Sleep and Movement Disorders University of Bonn Bonn Germany

**Keywords:** disease prognosis, prodromal dementia with Lewy bodies, prodromal disease definitions, prodromal Parkinson's disease, risk disclosure

## Abstract

**Background:**

Isolated REM sleep behavior disorder (iRBD) indicates an early α‐synucleinopathy with approximately 90% of individuals pheno‐converting to Parkinson's disease (PD), dementia with Lewy bodies (DLB), or multiple system atrophy (MSA). Recently, prodromal disease definitions (pPD, pDLB, pMSA) have been introduced.

**Objective:**

To investigate the overlap of prodromal definitions in an established iRBD cohort.

**Methods:**

We applied the current diagnostic criteria for pPD, pDLB, and pMSA to *N* = 55 individuals from the local iRBD cohort.

**Results:**

All except two individuals fulfilled at least one of the prodromal disease definitions; most individuals were classified as pPD (94.5%). 56% of the individuals fulfilled more than one definition: 32.7% pMSA&pPD, 10.9% pDLB&pPD, and 12.7% all three (pPD&pDLB&pMSA). The cognitive screening [(pPD = pMSA) > pDLB], motor symptoms [pPD < (pDLB = pMSA)], and olfactory testing [(pPD = pDLB) < pMSA] significantly differed between groups.

**Conclusions:**

The observed overlap leads to challenges in patient counseling and risk disclosure. Better discrimination will facilitate research in early α‐synucleinopathy phases.

Isolated rapid eye movement (REM) sleep behavior disorder (iRBD) represents a non‐REM parasomnia and indicates an early α‐synucleinopathy,^1^, ^2^ with approximately 90% of individuals developing clinically manifest Parkinson's disease (PD), dementia with Lewy bodies (DLB), or multiple system atrophy (MSA) within 15 years of iRBD diagnosis.^3^, ^4^ For all three entities, prodromal disease definitions have been proposed (pPD, pDLB, pMSA),^5^, ^6^, ^7^, ^8^ with the current pPD framework explicitly including DLB as a potential outcome.^5^, ^6^, ^9^

With the rise of α‐synuclein seed amplification assays (α‐syn‐SAA) allowing for in‐vivo detection of misfolded α‐synuclein, the path for biological definitions of α‐synucleinopathies was paved.^10^, ^11^ The proposed biological definitions unify what is currently defined as (p)PD and (p)DLB, excluding (p)MSA. Similarities between the two recently introduced definitions include an α‐synuclein biomarker and a neurodegeneration biomarker (eg, pathological dopamine transporter scan, DaTscan) as critical biological anchors and the clinical symptoms as an additional axis.^10^, ^11^ The assessment of shared clinical features (eg, iRBD, autonomic dysfunction, subthreshold parkinsonism), with further red flags or supportive criteria, enriches the biological α‐synuclein disease definition and may allow differentiating clinical subtypes.^10^, ^11^, ^12^

With iRBD constituting an essential clinical feature of all three prodromal disease definitions^5^, ^6^, ^7^, ^8^ and a pre‐defined overlap between pPD and pDLB, a significant overlap of prodromal disease classifications in individuals with iRBD is anticipated, limiting their use for differential disease prognosis for individuals with iRBD. Of note, the current prodromal disease definitions^5^, ^6^, ^7^, ^8^ were independently developed as research criteria for each prodromal entity. The objective of our study is to practically recapitulate “state‐of‐the‐art” clinical decision making of what disease entity is present using the published research prodromal criteria in the context of an existing iRBD cohort.

## Methods

### Study Design and Participants

The present cross‐sectional analyses are based on the baseline data of a subsample of our local polysomnography‐confirmed iRBD cohort^13^: We included individuals with at least one clinical consultation between July 2020 and November 2023 and a completed cognitive test battery suitable to assess mild cognitive impairment (MCI) with Level‐II criteria,^14^ (as a prerequisite for applying the pDLB criteria), resulting in a sample of *N* = 55 individuals for the present analyses (representing 50% of the total iRBD cohort, for details, see Supplementary Material S1). Details regarding the cognitive assessments are reported in the Supplementary Material 2. All subjects gave written informed consent before entering the study cohort's diagnostic workflow. The ethics committee of the Faculty of Medicine of the University of Cologne approved the study conducted under the Declaration of Helsinki.

### Operationalization of Prodromal Criteria

The current prodromal criteria were used to diagnose pPD, pDLB, and pMSA.^5^, ^6^, ^7^, ^8^ Details regarding the assessed prodromal features, including their operationalization, are reported in the Supplementary Material 3.

To summarize, pPD was diagnosed, if the prodromal disease definition for probable prodromal PD was fulfilled,^5^, ^6^ that is, if a probability of prodromal PD of ≥80% was reached in the likelihood ratio (LR) calculation, considering as many risk and prodromal markers as available. We assessed 8/10 risk markers and all prodromal markers. However, some markers were only assessed in a subset of individuals (eg, DaTscan).

pDLB was diagnosed, if the definition for probable MCI‐LB^7^ was fulfilled. MCI as the essential feature was defined by the presence of questionnaire‐based subjective cognitive decline (SCD), Level‐II objective cognitive decline,^14^ and preserved activities of daily living assessed by medical history. Polysomnography‐proven iRBD, parkinsonism, and recurrent visual hallucinations were assessed as core clinical features. REM sleep without atonia (RSWA) as a proposed biomarker was confirmed by polysomnography. Furthermore, a subset of participants underwent DaTscan. We assessed all essential features, 3/4 core clinical features, 2/3 proposed biomarkers, 12/13 supportive clinical features, however, none of the potential biomarkers.

pMSA was diagnosed, if the definition for possible prodromal MSA^8^ was fulfilled. A sporadic, progressive adult disease onset as the essential feature was assessed by medical history. Polysomnography‐proven iRBD and neurogenic orthostatic hypotension (OH) were assessed as the clinical non‐motor features, and subtle parkinsonian signs and subtle cerebellar signs as the clinical motor features. Unexplained anosmia as a mandatory exclusion criterion (as we did not perform cardiac sympathetic imaging, 123I‐MIBG‐scintigraphy) was operationalized by a Sniffin’ Sticks (Burkhardt®, Wedel, Germany) score <6. Sensitivity analyses additionally evaluated the cut‐off of <10 for hyposmia as an exclusion criterion for pMSA. We assessed the essential criterion, 2/3 proposed clinical non‐motor features, all clinical motor features, 1/2 potential mandatory exclusion criteria, and 5/6 further exclusion criteria.

### Statistical Analyses

Data were managed and analyzed using R.^15^ For each prodromal disease definition and combinations thereof, the percentages of positive diagnoses were reported. Kruskal‐Wallis tests with follow‐up Mann–Whitney‐*U* tests with clinical variables as dependent variables or Chi‐squared tests were conducted to analyze which clinical features drive the overlap between prodromal disease definitions. Alpha‐level for these comparisons was set to 0.05.

## Results

The individuals with iRBD were 69.41 ± 5.75 years old, 87.3% male, and reported a time of 8.02 ± 6.64 years since their first RBD reported symptoms. We identified objective MCI in 27.3% of individuals with iRBD, 31.5% were anosmic and 57.4% hyposmic according to olfactory testing, and 42.6% showed OH. Further sample characteristics are summarized in Table 1.

**TABLE 1:** Sample characteristics

			ALL	pPD*	pDLB*	pMSA*	*p*
			*N* = 55	*n* = 21	*n* = 13	*n* = 19	
Age			69.41 (5.75)	70.26 (5.31)	69.19 (6.27)	69.21 (6.07)	0.805
			[55.90–80.93]	[61.03–80.93]	[59.15–79.02]	[55.90–77.56]	
Sex	Female		7 (12.73%)	4 (19.05%)	2 (15.38%)	1 (5.26%)	0.422
	Male		48 (87.27%)	17 (80.95%)	11 (84.62%)	18 (94.74%)	
Years of education			15.62 (3.17)	16.21 (3.22)	13.54 (2.91)	16.13 (2.92)	0.045
			[7–22]	[10–21.50]	[7–19]	[11–22]	
Time since first reported RBD symptoms in years			8.02 (6.64)	8.31 (7.10)	6.20 (5.40)	9.06 (7.02)	0.482
			[1.43–28.94]	[1.43–28.94]	[1.77–20.13]	[1.71–27.38]	
Sniffin’ sticks			6.48 (2.55)	5.38 (2.25)	5.54 (2.70)	8.32 (1.73)	<0.001
			[0–12]	[0–9]	[0–10]	[6–12]	
Olfactory impairment	Anosmia		17 (31.48%)	12 (57.14%)	5 (38.46%)	0 (0%)	0.001
	Hyposmia		31 (57.41%)	9 (42.86%)	7 (53.85%)	14 (73.68%)	
Orthostatic hypotension			23 (42.59%)	9 (42.86%)	5 (38.46%)	9 (47.37%)	0.881
MDS‐UPDRS‐III			5.43 (3.08)	3.71 (2.33)	5.85 (3.16)	7.21 (2.78)	<0.001
			[0–13]	[0–9]	[2–12]	[4–13]	
MoCA			26.05 (2.07)	26.24 (1.95)	24.54 (1.56)	26.47 (1.84)	0.011
			[23–30]	[23–30]	[23–27]	[23–30]	
Level‐II cognitive impairment	a‐sd‐mci		2 (3.64%)	0 (0%)	2 (15.38%)	0 (0%)	<0.001
	a‐md‐MCI		10 (18.18%)	1 (4.76%)	9 (69.23%)	0 (0%)	
	na‐md‐MCI		3 (5.45%)	1 (4.76%)	2 (15.38%)	0 (0%)	
Subjectively impaired domains			1.47 (1.42)	1.20 (1.51)	2 (1.33)	1.53 (1.39)	0.257
			[0–5]	[0–5]	[0–4]	[0–4]	
BDI‐II			6.28 (7.35)	4.95 (8.89)	9.75 (6.59)	5.95 (5.74)	0.031
			[0–41]	[0–41]	[0–24]	[0–17]	
BAI			4.66 (5.51)	4.58 (6.38)	6.73 (5.33)	3.78 (4.82)	0.061
			[0–25]	[0–25]	[2–21]	[0–20]	
AES			27.92 (8.50)	27.15 (8.47)	33.73 (9.61)	25.94 (6.80)	0.088
			[17–50]	[17–44]	[18–50]	[17–39]	
pPD	Probability		0.96 (0.08)	0.97 (0.05)	0.98 (0.04)	0.96 (0.08)	0.555
			[0.57–1]	[0.84–1]	[0.85–1]	[0.74–1]	
pDLB	Essential	1	13 (23.64%)	0 (0%)	13 (100%)	0 (0%)	<0.001
	Core	1	24 (43.64%)	15 (71.43%)	3 (23.08%)	4 (21.05%)	0.002
		2	31 (56.36%)	6 (28.57%)	10 (76.92%)	15 (78.95%)	
	Biomarker	1	41 (74.55%)	16 (76.19%)	10 (76.92%)	13 (68.42%)	0.815
		2	14 (25.45%)	5 (23.81%)	3 (23.08%)	6 (31.58%)	
	Supportive	1	7 (12.73%)	0 (0%)	3 (23.08%)	3 (15.79%)	0.002
		2	28 (50.91%)	18 (85.71%)	3 (23.08%)	7 (36.84%)	
		3	13 (23.64%)	2 (9.52%)	6 (46.15%)	5 (26.32%)	
		4	1 (1.82%)	0 (0%)	1 (7.69%)	0 (0%)	
		5	1 (1.82%)	1 (4.76%)	0 (0%)	0 (0%)	
pMSA	Essential	1	55 (100%)	21 (100%)	13 (100%)	19 (100%)	n.a.
	Non‐motor	1	32 (58.18%)	12 (57.14%)	8 (61.54%)	10 (52.63%)	0.881
		2	23 (41.82%)	9 (42.86%)	5 (38.46%)	9 (47.37%)	
	Motor	0	20 (36.36%)	15 (71.43%)	3 (23.08%)	0 (0%)	<0.001
		1	35 (63.64%)	6 (28.57%)	10 (76.92%)	19 (100%)	
	Exclusion criteria	0	38 (69.81%)	9 (42.86%)	8 (61.54%)	19 (100%)	<0.001
		1	17 (30.91%)	12 (57.14%)	5 (38.46%)	0 (0%)	

*Note*: Data are mean (standard deviation) [range: minimum‐maximum] or n (%). P‐values of Kruskal‐Wallis tests or Chi‐squared tests are reported as appropriate.

Abbreviations: AES, Apathy evaluation scale; a‐md‐MCI, amnestic multi‐domain mild cognitive impairment; a‐sd‐MCI, amnestic single‐domain mild cognitive impairment; BAI, Beck Anxiety Inventory; BDI‐II, Beck Depression Inventory; MDS‐UPDRS‐III, Movement Disorder Society Unified Parkinson's Disease Rating Scale Part 3; MoCA, Montréal Cognitive Assessment; na‐md‐MCI, non‐amnestic multi‐domain mild cognitive impairment; pDLB, prodromal dementia with Lewy bodies; pMSA, prodromal multiple system atrophy; pPD, prodromal Parkinson's disease; RBD, rapid eye movement behavior disorder.

* Groups were created so that each individual is represented only once. The pPD group includes those fulfilling the pPD criteria only, the pDLB group includes those fulfilling pDLB&pPD and pDLB&pPD&pMSA, and the pMSA group pMSA and pMSA&pPD. Two individuals did not fulfill any of the three prodromal disease definitions.

**Figure 1:**
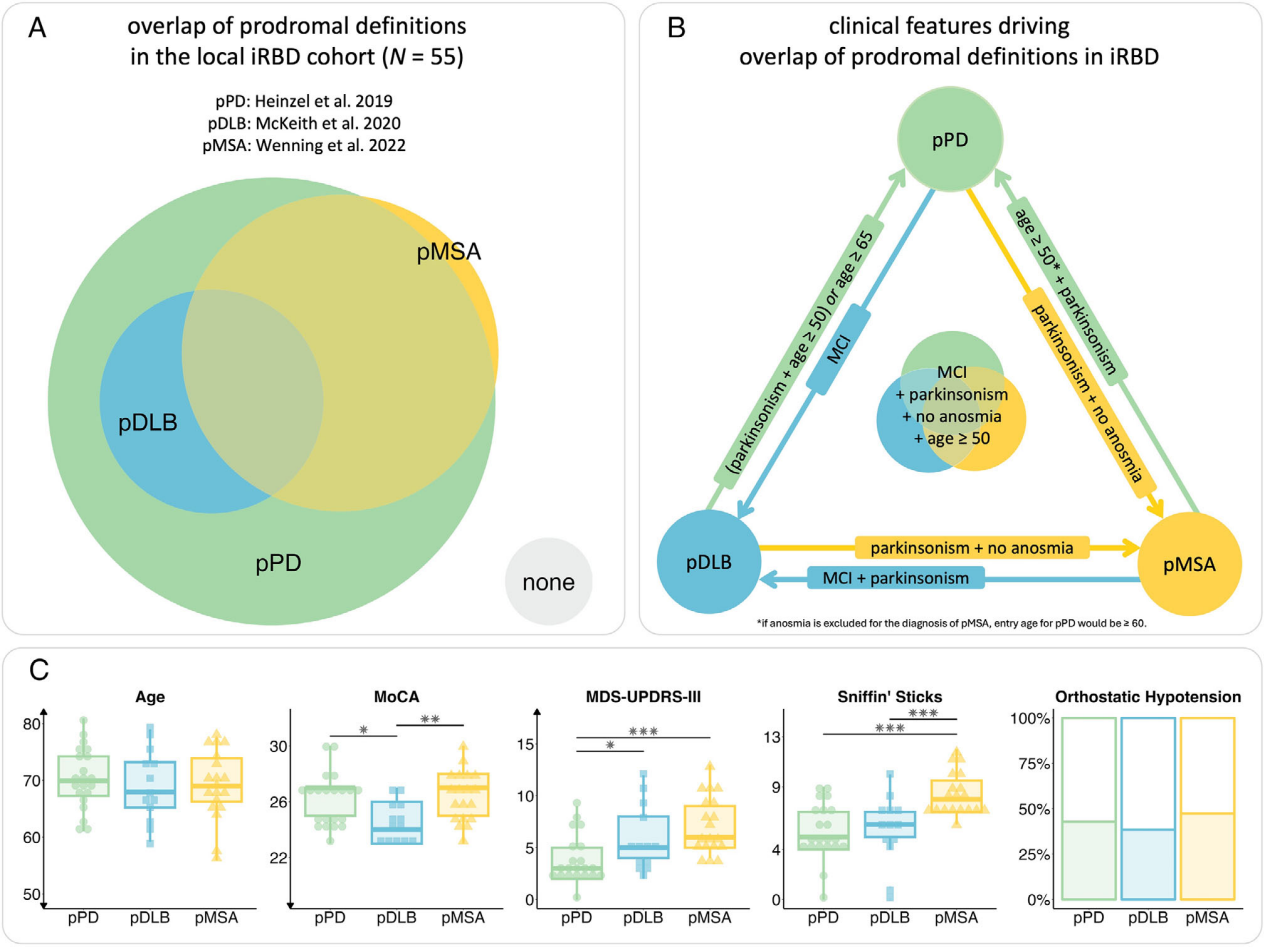
(**A**) Overlap of definitions for prodromal Parkinson's disease (pPD, green), prodromal dementia with Lewy bodies (pDLB, blue), and prodromal multiple system atrophy (pMSA, yellow) in our iRBD cohort (*N* = 55). The area‐proportional Euler diagram was fitted using the *eulerr* R‐package (https://CRAN.R‐project.org/package=eulerr). (**B**) Conceptual framework of the clinical features driving the overlap of prodromal disease definitions when iRBD is present. Indicated age in years represents the age required to reach a probability of ≥80% for a diagnosis of probable pPD given the pre‐defined prodromal factors according to the revised MDS criteria for prodromal PD (3). MCI, mild cognitive impairment. (**C**) Age in years, MoCA (Montréal Cognitive Assessment) scores, MDS‐UPDRS‐III (Unified Parkinson's Disease Rating Scale Part 3) scores, Sniffin’ Sticks score and presence of orthostatic hypotension in percent of individuals fulfilling the prodromal disease definitions of pPD (ie, pPD only, green), pDLB (ie, pDLB&pPD and pDLB&pPD&pMSA, blue), and pMSA (ie, pMSA and pMSA&pPD, yellow). Dots/squares/triangles represent individual scores. The group‐wise boxplots visualize the within‐group median, the hinges represent the corresponding first and the third quartile, and the whiskers are 1.5 times the interquartile range. Asterisks represent the significance of pairwise Mann–Whitney U tests, ^+^ *p* < .100, *p < .050, ** *p* ≤ .010, *** *p* ≤ .001.

At least one of the prodromal disease definitions was fulfilled in 96.4% of the individuals with iRBD, and 94.5% could be classified as pPD. “pPD only” was fulfilled in 38.2% of individuals, “pMSA only” in 1.8%. In contrast, all individuals with pDLB fulfilled at least one additional prodromal definition. Overall, 56.4% fulfilled more than one definition: 32.7% were classified as pMSA&pPD, 10.9% as pDLB&pPD, and 12.7% fulfilled all three prodromal definitions (pPD&pDLB&pMSA). The overlap is visualized in Figure 1A.

To analyze which clinical features drive the overlap or differentiate between prodromal disease definitions (Table 1 and Fig. 1C), we compared three groups: individuals with pPD only (ie, “pPD only”), individuals with pDLB in any combination (ie, pDLB&pPD and pDLB&pPD&pMSA), and individuals with pMSA only or in combination with pPD (ie, “pMSA only” and pMSA&pPD). Montréal Cognitive Assessment (MoCA)^16^ scores [*χ* ^2^(2) = 8.98, *p* = .011, (pPD = pMSA) > pDLB], Movement Disorder Society Unified Parkinson's Disease Rating Scale Part 3 (MDS‐UPDRS‐III)^17^ total scores [*χ* ^2^(2) = 15.721, *p* < 0.001, pPD < (pDLB = pMSA)], and the Sniffin’ Sticks score [*χ* ^2^(2) = 16.92, *p* < 0.001, (pPD = pDLB) < pMSA] significantly differed between groups. Sample characteristics and statistics including individuals fulfilling the prodromal definition for pPD, pMSA, pDLB in any combination (ie, individuals being represented in multiple groups as applicable) are reported in the Supplementary Material 4. Supplementary Material 5 reports the results of the sensitivity analyses with hyposmia rather than anosmia as an exclusion criterion for pMSA.

## Discussion

By applying the current prodromal disease definitions of pPD, pDLB, and pMSA,^5^, ^6^, ^7^, ^8^ we observed a substantial overlap of prodromal disease definitions in a cohort of individuals with iRBD. We identified cognitive status, subthreshold parkinsonism, and olfactory functioning as the most critical features that can drive this overlap depending on the context, as their presence or absence does not lead to a dichotomous outcome. Our data suggest that a higher specificity of definitions is needed, if they are to be applied to support patient counseling, as pathways of iRBD disease progression are highly heterogeneous.^18^

Polysomnography‐proven isolated RBD is the most critical clinical marker within all current prodromal disease definitions and should, therefore, always be included in the diagnostic workflow. The most recent multicenter data analysis by the International RBD Study Group (IRBDSG) revealed that 58.6% of the individuals with iRBD convert to parkinsonism‐first (including both PD, 53.6%, and MSA, 5%) and 41.4% developed DLB.^3^ IRBD is the prodromal marker with the highest LR for pPD (LR_iRBD_ = 130).^6^ Therefore, even without the presence of any other prodromal or risk marker, any individual with iRBD above the age of 75 fulfills the pPD criteria, with additional MCI decreasing this threshold to 65 years and additional subthreshold parkinsonism to 50 years (Fig. 1B). It has been suggested that iRBD is crucial for the sensitivity of the current pPD criteria.^12^ However, iRBD is only present in around one‐quarter of individuals before manifest PD.^19^, ^20^ IRBD is also a core clinical feature for pDLB: all individuals with iRBD, based on the ICSD‐III criteria, and additional MCI qualify for pDLB.^7^ For diagnosing pMSA, only one additional motor feature and excluding anosmia or an abnormal MIBG is mandatory when iRBD is diagnosed.^8^

Particularly, the prevalence of pMSA is likely to be overestimated with the current criteria^8^ in individuals with iRBD. So far, the prodromal disease definitions focus on maximizing sensitivity by rather unspecific key clinical features, while further supportive and exclusion criteria bear the potential to increase their specificity. This holds specifically true for the definition of olfactory impairment in pMSA, for example, by considering hyposmia as olfactory impairment rather than more advanced anosmia (see sensitivity analyses reported in Supplementary Material 5).

The absence of 123I‐MIBG scintigraphy in our diagnostic workflow is a major limitation of this study and may contribute to an overestimation of the pMSA prevalence.^8^, ^21^, ^22^ However, it has to be mentioned that a normal 123I‐MIBG scan might also occur in pPD within the framework of a body‐ or brain‐first subtyping.^23^ The criterion for parkinsonism in each of the prodromal criteria, sometimes explicitly defined by a specific UPDRS‐III threshold, still introduces an element of subjectivity.^24^ In this context, the potential for more objective markers of early motor changes—such as those assessed by wearable devices^25^ or automated, video‐based scoring pipelines^26^—should be acknowledged and further evaluated in the future. Notably, neither exclusion criteria nor ranking markers concerning core or essential criteria exist for pPD: each assessed marker simply increases or decreases the likelihood for pPD.^5^, ^6^ Furthermore, the pPD criteria include pDLB cases by definition.^5^, ^6^, ^9^ Therefore, if applied separately from the pDLB criteria, no conclusions regarding differential prognosis should be drawn.

In our cohort, only about one‐quarter of the individuals with iRBD fulfilled the criteria for pDLB. In contrast, about 40% of them are expected to develop a dementia‐first syndrome.^3^ The observation that cognitive impairment may potentially occur relatively late in the course of pDLB could therefore be easily (mis)interpreted as a lack of sensitivity of the pDLB criteria.^7^ Our cohort is generally highly educated, which may have contributed to the potential underidentification of MCI. Furthermore, investigating the role of SCD as a potential prodrome of MCI in α‐synucleinopathies may refine our understanding of the dementia‐first pathway.^27^, ^28^

The possibility of α‐syn‐SAA detection is a milestone in early diagnosis and potential early disease‐modifying treatment initiation. However, it will never discharge from a thorough phenotyping of those affected. Currently, α‐syn‐SAA are not capable of confidently differentiating between pPD, pDLB, and pMSA,^29^, ^30^ and the availability of α‐synuclein detection and imaging biomarkers is limited for population‐based studies. Early clinical symptoms will remain the primary reason for clinical consultations and can be used to build enriched cohorts undergoing biological characterization. Integrating biological and clinical disease characterizations will refine our understanding of the clinical heterogeneity and potential shared pathways in early phases, including our understanding of co‐pathology to Alzheimer's disease.^31^ Further, it will enhance the specificity of disease prognosis. A mutual interplay between developments in biological disease definitions and detailed phenotyping in the early phase of α‐synucleinopathies is warranted.

In the IRBDSG analysis, the only reliable clinical markers differentiating between the pPD and pDLB pathways were the higher prevalence of cognitive impairment and the faster decline in cognitive abilities in DLB‐converters,^3^ which may support the inclusion of longitudinal features in revised prodromal disease definitions. The field of research in early α‐synucleinopathies may benefit from recommendations for a minimum set of standardized assessments and guidelines, how clinical features beyond core features may be used for patient counseling, risk disclosure, family support, and symptomatic management regarding all potential disease pathways.^10^ In that sense, a singular, overarching prodromal definition encompassing all currently separated definitions may be superior and better account for the different clinical outcomes. Individual symptom profiles may also guide personalized lifestyle counseling for secondary prevention.

Diagnosing iRBD is accompanied by the ethical challenge of adequately disclosing the implications of the diagnosis to affected individuals, with the majority of individuals with iRBD asking for their prognosis.^18^ To date, the current prodromal disease definitions^5^, ^6^, ^7^, ^8^ may represent the closest available approach to a differential diagnostic tool; however, they were independently developed as research criteria for each entity and not designed as a framework for differential diagnosis and patient counseling. For a meaningful and effective use of the prodromal criteria in a clinical context, focusing on the affected individuals, we need to refine our current prodromal disease definitions.

## Author Roles

(1) Research project: A. Conception, B. Organization, C. Execution; (2) Statistical analysis: A. Design, B. Execution, C. Interpretation, D. Review and critique; (3) Manuscript: A. Writing of the first draft, B. Review and critique.

A.O.: 1A, 1B, 1C, 2A, 2B, 2C, 3A.

S.R.: 1B, 1C, 2D, 3B.

J.R.: 1C, 2D, 3B.

E.K.: 2D, 3B.

G.R.F.: 1A, 2D, 3B.

M.S.: 1A, 1B, 1C, 2C, 2D, 3A.

## Disclosures

**Funding Sources and Conflict of Interest:** AO received grants from the Koeln Fortune Program (grant‐no. 329/2021, 142/2023), Faculty of Medicine, University of Cologne, and the “Novartis‐Stiftung für therapeutische Forschung”. MS received grants from the Else Kröner‐Fresenius‐Stiftung (grant number 2019_EKES.02). MS is receiving funding from the program “Netzwerke 2021”, an initiative of the Ministry of Culture and Science of the State of Northrhine Westphalia. JR, SR, EK, GRF declare that there are no conflicts of interest relevant to this work.

**Financial Disclosures for the previous 12 months:** AO, JR, SR, MS declare that there are no additional disclosures to report. EK received grants from the German Ministry of Education and Research, the Joint Federal Committee, and The German Parkinson Foundation, all outside the submitted work. EK received honoraria from the companies EISAI GmbH, Germany, memodio GmbH, Germany, Desitin GmbH, Germany, and Prolog GmbH, Germany, all outside the submitted work. GRF serves as an editorial board member of Cortex, Neurological Research and Practice, NeuroImage: Clinical, Zeitschrift für Neuropsychologie, and Info Neurologie & Psychiatrie; receives royalties from the publication of the books Funktionelle MRT in Psychiatrie und Neurologie, Neurologische Differentialdiagnose, SOP Neurologie, and Therapiehandbuch Neurologie; receives royalties from the publication of the neuropsychological tests KAS and Köpps; received honoraria for speaking engagements from Deutsche Gesellschaft für Neurologie (DGN) and Forum für medizinische Fortbildung FomF GmbH; receives funding from the Deutsche Forschungsgemeinschaft (CRC 1451; Project‐ID 431549029).

**Ethical Compliance Statement:** The ethics committee of the Faculty of Medicine of the University of Cologne approved the study conducted under the Declaration of Helsinki. All subjects gave written informed consent before entering the study cohort's diagnostic workflow. We confirm that we have read the Journal's position on issues involved in ethical publication and affirm that this work is consistent with those guidelines.

## Supporting information


**Supplementary Material S1.** Comparison between individuals in the iRBD cohort only and those with a completed cognitive test battery suitable to assess mild cognitive impairment (MCI) with Level‐II criteria.
**Supplementary Material S2.** Details and references of the clinical assessments and cognitive test battery to assess mild cognitive impairment with Level‐II criteria.
**Supplementary Material S3.** Operationalization of prodromal criteria in the present study.
**Supplementary Material S4.** Sample characteristics and statistics including individuals fulfilling the prodromal definition in any combination (ie, individuals being represented in multiple groups as applicable).
**Supplementary Material S5.** Sensitivity analyses excluding hyposmia rather than anosmia for prodromal multiple system atrophy.

## Data Availability

The data that support the findings of this study are available on request from the corresponding authors. The data are not publicly available due to privacy or ethical restrictions.
