# Supplementary material for: Being Prodromal: Current Criteria in the Context of Isolated REM Sleep Behavior Disorder
Source: Mov Disord Clin Pract. 2025 Feb 22;12(6):828–34. doi: 10.1002/mdc3.70006 (PMC12187958; doi:10.1002/mdc3.70006)
Supplement: Supplementary file 1 — Supplementary Material S1. Comparison between individuals in the iRBD cohort only and those with a completed cognitive test battery suitable to assess mild cognitive impairment (MCI) with Level‐II criteria. Supplementary Material S2. Details and references of the clinical assessments and cognitive test battery to assess mild cognitive impairment with Level‐II criteria. Supplementary Material S3. Operationalization of prodromal criteria in the present study. Supplementary Material S4. Sample characteristics and statistics including individuals fulfilling the prodromal definition in any combination (ie, individuals being represented in multiple groups as applicable). Supplementary Material S5. Sensitivity analyses excluding hyposmia rather than anosmia for prodromal multiple system atrophy. [file MDC3-12-828-s001.pdf]

SUPPLEMENTARY MATERIAL 1 – 5

Being prodromal:

Current prodromal criteria in the context of isolated REM sleep behavior disorder

---

Supplementary Material 1. Comparison between individuals in the iRBD cohort only and those with a completed cognitive test battery suitable to assess mild cognitive impairment (MCI) with Level-II criteria.

Supplementary Material 2. Details and references of the clinical assessments and cognitive test battery to assess mild cognitive impairment with Level-II criteria.

Supplementary Material 3. Operationalization of prodromal criteria in the present study.

Supplementary Material 4. Sample characteristics and statistics including individuals fulfilling the prodromal definition in any combination (i.e., individuals being represented in multiple groups as applicable).

Supplementary Material 5. Sensitivity analyses excluding hyposmia rather than anosmia for prodromal multiple system atrophy.

SUPPLEMENTARY MATERIAL 1

Comparison between individuals in the iRBD cohort only and those with a completed cognitive test battery suitable to assess mild cognitive impairment (MCI) with Level-II criteria.

		Cohort only (*n* = 55)	BeingProdromal (*n* = 55)	Mann-Whitney U test
**Age**		66.3 (7.47)	68.28 (5.84)	*U* = 1256, *p* = .165, |*r*| = 0.13
**Sex**	female	9 (16.4%)	7 (12.7%)	$\chi^2(1)$=0.07, *p* = .786
	male	46 (83.6%)	48 (87.3%)	
**Time since first reported RBD symptoms in years**		7.93 (5.56)	8.02 (6.64)	*U* = 1356, *p* = .582, |*r*| = 0.05
**Sniffin' Sticks**		7.19 (2.6)	6.48 (2.55)	*U* = 1673.5, *p* = .182, |*r*| = 0.13
**MDS-UPDRS-III**		4.96 (3.53)	5.43 (3.08)	*U* = 1330.5, *p* = .346, |*r*| = 0.09
**BDI-II**		7.78 (9.62)	6.28 (7.35)	*U* = 1448.5, *p* = .52, |*r*| = 0.06
**BAI**		5.4 (6.53)	4.66 (5.51)	*U* = 1251, *p* = .994, |*r*| = 0
**AES**		29.46 (8.68)	27.92 (8.5)	*U* = 1416, *p* = .338, |*r*| = 0.09

Notes. AES, Apathy Evaluation Scale; BAI, Beck Anxiety Inventory; BDI-II, Beck Depression Inventory; RBD, rapid eye movement behavior disorder; MDS-UPDRS-III, Movement Disorder Society Unified Parkinson's Disease Rating Scale Part 3.

SUPPLEMENTARY MATERIAL 2

Clinical Assessments and Cognitive Test Battery

Demographic characteristics, clinical rating scales (MDS Unified Parkinson's Disease Rating Scale, MDS-UPDRS[1], Unified Multiple System Atrophy Rating Scale, UMSARS[2], Orthostatic hypotension test: 10 min lying, followed by 3 min standing[3]), and olfactory functioning with Sniffin' Sticks (Burkhardt®, Wedel, Germany) were assessed during a clinical visit by trained psychologists and neurologists. Questionnaires were filled out following the clinical visit digitally or on paper: SCales for Outcomes in PArkinson's disease - Autonomic Dysfunction (SCOPA-AUT)[4], Epworth Sleepiness Scale (ESS)[5], Beck Depression Inventory (BDI-II)[6], Beck Anxiety Inventory (BAI)[7], Apathy Evaluation Scale (AES)[8], Mild Behavioral Impairment Checklist (MBI-C)[9], Multi-domain Subjective Cognitive Decline Evaluation (Multi-SubCoDE)[10]. A subset of participants underwent DaTscan and a clinical 1.5 Tesla MRI scan.

The cognitive assessment including the Montréal Cognitive Assessment (MoCA, [11]) and a Level-II cognitive battery for mild cognitive impairment (MCI)[12] was administered during a separate visit. At least two tests per five cognitive domains (executive functions, attention and working memory, memory, visuo-cognition, language) were assessed. Objective evidence of impairment as required for the pDLB operationalization[13] was defined as the presence of impaired test performance ≤-1 SD below published normative data in at least two tests within one or more of the investigated cognitive domains[12]. The cognitive test battery including references of tests as well as an indication of whether the tests were used for the Level-II assessment of MCI is reported in the Table below. If two test scores of the same test (e.g., immediate and delayed recall of a word list) are impaired, another test needs to be impaired to meet the criterion of two test abnormalities.

DOMAIN	Abbreviation	Assessment	Relevant for Level-II MCI Assessment	Reference
**COGNITION**				
**Overall Cognitive State**				
Subjective Cognition	Multi-SubCoDE	Multi-Domain Subjective Cognitive Decline Evaluation		10
Global Cognition	MoCA	Montreal Cognitive Assessment		11
**Executive**				
Semantic fluency	RWT sem	Semantic Verbal Fluency: Food, Regensburger Wortflüssigkeitstest	X*	14
Phonemic fluency	RWT phon	Phonemic Verbal Fluency: P-Words, Regensburger Wortflüssigkeitstest	X*	14
Set-Shifting	TMTB/A	Trail Making Test (TMT): TMT-B / TMT-A	X	15; 16
**Visuo-cognition**				
Construction	ROCFT	Rey Osterrieth Complex Figure Test (ROCFT): Figure Copy	X	17,18
Perception	LPS-11	Visual Perception, Leistungsprüfsystem 50+: Subtest 11	X*	19
Spatial Perception	LPS-7	Spatial Rotation, Leistungsprüfsystem 50+: Subtest 7	X*	19
**Attention & Working Memory**				
Working Memory	DSback	Digit Span backwards, Wechsler Adult Intelligence Scale (WAIS)	X	20
Processing Speed	TMT-A	Trail Making Test A	X	15; 16
**Memory**				
Verbal Memory	VLMT-Learn	Wordlist Learning, Verbaler Lern- und Merkfähigkeitstest (VLMT)	X*	21
	VLMT-Rec	Wordlist Recall, Verbaler Lern- und Merkfähigkeitstest (VLMT)	X*	21
Visuo-spatial Memory	ROCFT	Rey Osterrieth Complex Figure Test (ROCFT): Figure Recall	X	17,18
**Language**				
Naming	ACL-Naming	Aphasia Check List, Subtest Naming	X	22
Semantic and Abstraction	WAIS	Similarities, Wechsler Adult Intelligence Scale (WAIS)	X	23

SUPPLEMENTARY MATERIAL 3

Operationalization of Prodromal Criteria

Prodromal PD

pPD was diagnosed, if the prodromal disease definition for probable prodromal PD was fulfilled[24,25], i.e., if a probability of prodromal PD of ≥ 80% was reached according to the likelihood ratio (LR) calculation, considering as many risk and prodromal markers as available. We assessed 8 of 10 risk markers and all prodromal markers.

Probable prodromal PD

Berg et al. 2015	https://doi.org/10.1002/mds.26431	24
Heinzel et al. 2019	https://doi.org/10.1002/mds.27802	25

≥ 80 % certainty
previous probability based on age
as many risk and prodromal markers as available

Domain	*Feature*	*Operationalization*
**Prior**	age	age in years
**Risk markers**	male sex	sex: male / female
	regular pesticide exposure (i.e., ≥100 episodes of non-occupational exposure)	Clinical visit (explicit question)
	occupational solvent exposure	Clinical visit (explicit question)
	nonuse of caffeine (i.e., <3 cups of coffee/6 cups of black tea per-week)	Clinical visit (explicit question)
	current smoker	Clinical visit (explicit question)
	never smoker	Clinical visit (explicit question)
	former smoker	Clinical visit (explicit question)
	first-degree relative with PD *OR*	Clinical visit (explicit question)
	known gene mutation *OR*	n.a.
	polygenic risk score	n.a.
	SN hyperechogenicity	n.a.
	Diabetes mellitus (type II)	Clinical visit (explicit question)
	physical inactivity	Clinical visit (explicit question)
	low plasma urate levels	n.a.
**Prodromal markers**	**polysomnography-proven RBD** *OR*	according to ICSD-3 [26]: RSWA + anamnestic or video-PSG acting out
	possible RBD (questionnaire)	n.a.
	Dopaminergic PET/SPECT clearly abnormal	DaTSCAN: ≥1 region of the basal ganglia showing reduced DaT uptake ≤-2 SD below age-corrected normative values
	Subthreshold parkinsonism *OR*	MDS-UPDRS-III >6 excluding postural and action tremor [1]
	Abnormal quantitative motor testing	n.a.
	Olfactory loss	Sniffin' Sticks (Burkhardt®, Wedel, Germany) <10
	Constipation	SCOPA-AUT ≤ 1x/2days, SCOPA-AUT 5+6 >1 OR medication [4]
	Excessive daytime somnolence	Epworth Sleepiness Scale total score ≥ 10 [5]
	Orthostatic hypotension (OH) – neurogenic OH *OR*	n.a.
	Symptomatic OH	Orthostatic hypotension test (10 min lying, 3 min standing) [3] singular systolic blood pressure drop ≥20 mmHg or a diastolic blood pressure drop ≥10mmHg upon standing compared to any supine blood pressure measurement [3]
	Erectile dysfunction	Clinical visit (explicit question)
	Urinary dysfunction	SCOPA-AUT (8+9+10+12) ≥ 2 [4]
	Depression (± anxiety)	Beck Depression Inventory ≥ 20 [6]
	Global cognitive deficit	≥ 2 tests ≤ -1.5 SD below the norm in a neuropsychological test battery with≥ 2 tests per five domains [12]

Notes. Non-assessed risk and prodromal markers are printed in grey font color and marked with n.a.. DaTSCAN, dopamine transporter (DaT) scan; ICSD-3, International Classification of Sleep Disorders III (ICSD-III) criteria; MDS-UPDRS-III, Movement Disorder Society Unified Parkinson's Disease Rating Scale Part 3; PSG, polysomnography; RSWA, rapid eyemovement sleep without atonia; SCOPA-AUT, SCales for Outcomes in PArkinson's disease - Autonomic Dysfunction

SUPPLEMENTARY MATERIAL 3 (continued)

Prodromal DLB

pDLB was diagnosed, if the definition for probable MCI-LB[13] was fulfilled. MCI as the essential feature was defined by the presence of questionnaire-based subjective cognitive decline, Level-II objective cognitive decline as described above[12], and preserved activities of daily living assessed by medical history. Polysomnography-proven iRBD, Parkinsonism, and recurrent visual hallucinations were assessed as the core clinical features. For all individuals with iRBD, REM sleep without atonia (RSWA) as a proposed biomarker was confirmed by polysomnography. Furthermore, a subset of participants underwent DaTscan. Reduced DaT uptake in basal ganglia was defined as ≥1 region of the basal ganglia showing reduced DaT uptake ≤-2 SD below age-corrected normative values. We assessed all essential features, 3 of 4 core clinical features, 2 of 3 proposed biomarkers, 12 of 13 supportive clinical features, however, none of the potential biomarkers.

Probable MCI-LB

McKeith et al. 2020 https://doi.org/10.1212/WNL.0000000000009323 [13]

Mild Cognitive Impairment

≥ 2 core clinical features OR

1 core clinical feature + ≥ 1 proposed biomarker

Domain	*Feature*	*Operationalization*
**essential**	subjective cognitive decline	Multi-SubCoDE ≥ 1 domain subjectively impaired [10]
	Level-II MCI	≥ 2 tests ≤ -1.5 SD below the norm in a neuropsychological test battery with≥ 2 tests per five domains [12]
	preserved activities of daily living	by medical history
core clinical features	Fluctuating cognition with variations in attention and alertness	n.a.
	Recurrent visual hallucinations	MDS-UPDRS-I Item 2 ≥ 3 [1]
	**polysomnography-proven RBD**	according to ICSD-3 [26]: RSWA + anamnestic or video-PSG acting out
	Parkinsonism	MDS-UPDRS-III: ≥ 1 subscore bradykinesia (3.4-3.8 + 3.14), rest tremor (3.17, 3.18), rigidity (3.3) ≥ 3 [1]
**proposed biomarkers**	Reduced dopamine transporter (DaT) uptake in basal ganglia demonstrated by SPECT or PET	DaTSCAN: ≥1 region of the basal ganglia showing reduced DaT uptake ≤-2 SD below age-corrected normative values
	PSG-confirmed RSWA	PSG-confirmed RSWA
	Reduced MIBG uptake on myocardial scintigraphy	n.a.
**supportive clinical features**	severe sensitivity to antipsychotic agents	by medical history
	postural instability	MDS-UPDRS-III 3.12 ≥ 2 [1]
	repeated falls	UMSARS 1.8 ≥ 2 [2]
	syncope or other transient episodes of unresponsiveness	SCOPA-AUT item 16 fainting ≥ 1 [4]
	prolonged or recurrent delirium	medical history
	autonomic dysfunction<br>e.g., constipation, orthostatic hypotension, urinary incontinence	Orthostatic hypotension test (10 min lying, 3 min standing) [3]; UMSARS 1.10, SCOPA-AUT ≤ 1x/2days, SCOPA-AUT 5+6 >1 OR medication [4]
	hypersomnia	n.a.
	hyposmia	Sniffin' Sticks (Burkhardt®, Wedel, Germany) <10
	hallucinations in other modalities	MDS-UPDRS-I 1.2 [1]
	systematized delusions	MBI-C domain: conviction and impression ≥ 3 [9]
	apathy	Apathy Evaluation Scale ≥ 34 [8]
	anxiety	Beck Anxiety Inventory ≥ 16 [7]
	depression	Beck Depression Inventory ≥ 20 [6]
**potential biomarkers**	EEG slowing	n.a.
	preservation of medial temporal lobe structures	n.a.
	Insular thinning and gray matter volume loss	n.a.

Notes. Non-assessed risk and prodromal markers are printed in grey font color and marked with n.a.. DaTSCAN, dopamine transporter (DaT) scan; ICSD-3, International Classification of Sleep Disorders III (ICSD-III) criteria; MDS-UPDRS, Movement Disorder Society Unified Parkinson's Disease Rating Scale; MBI-C, Mild Behavioral Impairment Checklist; MIBG, meta-iodobenzylguanidine; Multi-SubCoDE, Multi-Domain Subjective Cognitive Decline Evaluation; PSG, polysomnography; RSWA, rapid eyemovement sleep without atonia; SCOPA-AUT, SCales for Outcomes in PArkinson's disease - Autonomic Dysfunction; UMSARS, Unified Multiple System Atrophy Rating Scale

SUPPLEMENTARY MATERIAL 3 (continued)

Prodromal MSA

pMSA was diagnosed, if the definition for possible prodromal MSA[27] was fulfilled. A sporadic, progressive adult disease onset as the essential feature was assessed by medical history. Polysomnography-proven iRBD and neurogenic orthostatic hypotension (OH) were assessed as the clinical non-motor features, and subtle parkinsonian signs and subtle cerebellar signs as the clinical motor features. Unexplained anosmia as a mandatory exclusion criterion (as we did not perform cardiac sympathetic imaging, 123I-MIBG-scintigraphy) was operationalized by a Sniffin' Sticks (Burkhardt®, Wedel, Germany) score ≤ 6. We assessed the essential criterion, 2 of 3 proposed clinical non-motor features, all clinical motor features, 1 of 2 potential mandatory exclusion criteria, and 5 of 6 further exclusion criteria.

Possible prodromal MSA
Wenning et al. 2022 https://doi.org/10.1002/mds.29005 [27]

sporadic, progressive adult onset disease
≥ 1 clinical non-motor feature
≥ 1 clinical motor features
none of exclusion criteria

Domain	*Feature*	*Operationalization*
**essential**	A sporadic, progressive adult (>30 years) onset disease	by medical history
**clinical non-motor features**	**polysomnography-proven RBD**	according to ICSD-3 [26]: RSWA + anamnestic or video-PSG acting out
	neurogenic orthostatic hypotension	Orthostatic hypotension test (10 min lying, 3 min standing) [3] singular systolic blood pressure drop ≥20 mmHg or a diastolic blood pressure drop ≥10mmHg upon standing compared to any supine blood pressure measurement [3]
	urogenital failure	n.a.
**clinical motor features**	subtle parkinsonian signs	MDS-UPDRS-III >6 excluding postural and action tremor [1]
	subtle cerebellar signs	sum ≥ 3: UMSARS 2.3 (ocular motor dysfunction), UMSARS 2.10 (heel-knee test), UPDRS-III 3.16 finger-nose test
**exclusion criteria**	unexplained anosmia on olfactory testing OR	Sniffin' Sticks (Burkhardt®, Wedel, Germany) <6<br><br>*for sensitivity analyses, we repeated the analyses with the cut-off for hyposmia (instead of anosmia) in the Sniffin' Sticks examination <10, see Supplementary Material 4*
	abnormal cardiac sympathetic imaging (123I-MIBG- scintigraphy)	n.a.
	Fluctuating cognition with early decline in visuoperceptual abilities	n.a.
	Recurrent visual hallucinations	MDS-UPDRS-I Item 2 ≥ 3 [1]
	Dementia	by medical history
	Downgaze supranuclear palsy	by medical history
	Brain MRI findings suggestive of an alternative diagnosis	MRI
	Documentation of an alternative condition known to produce autonomic failure, ataxia, or parkinsonism and plausibly connected to the patient's symptoms	by medical history

Notes. Non-assessed risk and prodromal markers are printed in grey font color and marked with n.a.. ICSD-3, International Classification of Sleep Disorders III (ICSD-III) criteria; MDS-UPDRS, Movement Disorder Society Unified Parkinson's Disease Rating Scale; MIBG, meta-iodobenzylguanidine; MRI, magnetic resonance imaging; PSG, polysomnography; RSWA, rapid eyemovement sleep without atonia; UMSARS, Unified Multiple System Atrophy Rating Scale

SUPPLEMENTARY MATERIAL 4

Sample characteristics irrespective of overlap between disease definitions

			ALL<br>*N* = 55	pPD<br>*n* = 52	pDLB<br>*n* = 13	pMSA<br>*n* = 26
**Age**			69.41 (5.75)<br>[55.90 - 80.93]	69.69 (5.77)<br>[55.90 - 80.93]	69.19 (6.27)<br>[59.15 - 79.02]	69.55 (5.85)<br>[55.90 - 78.48]
**Sex**	female		7 (12.73%)	7 (13.46%)	2 (15.38%)	2 (7.69%)
	male		48 (87.27%)	45 (86.54%)	11 (84.62%)	24 (92.31%)
**Years of Education**			15.62 (3.17)<br>[7 - 22]	15.51 (3.22)<br>[7 - 22]	13.54 (2.91)<br>[7 - 19]	15.42 (3.37)<br>[7 - 22]
**RBD duration in years**			8.02 (6.64)<br>[1.43 – 28.94]	7.93 (6.68)<br>[1.43 - 28.94]	6.20 (5.40)<br>[1.77 - 20.13]	8.38 (6.82)<br>[1.71 - 27.38]
**Sniffin' Sticks**			6.48 (2.55)<br>[0 - 12]	6.40 (2.55)<br>[0 - 12]	69.19 (6.27)<br>[59.15 - 79.02]	8 (1.72)<br>[6 - 12]
**Olfactory impairment**	anosmia		17 (31.48%)	17 (32.69%)	5 (38.46%)	8 (1.72) [6 - 12]
	hyposmia		31 (57.41%)	30 (57.69%)	7 (53.85%)	0 (0%)
**MDS-UPDRS-III**			5.43 (3.08)<br>[0 - 13]	5.52 (3.10)<br>[0 - 13]	5.85 (3.16)<br>[2 - 12]	7.19 (2.84)<br>[4 - 13]
**MoCA**			26.05 (2.07)<br>[23 - 30]	25.87 (1.95)<br>[23 - 30]	24.54 (1.56)<br>[23 - 27]	25.92 (1.98)<br>[23 - 30]
**Level-II cognitive impairment**	a-sd-MCI		2 (3.64%)	2 (3.85%)	2 (15.38%)	2 (7.69%)
	a-md-MCI		10 (18.18%)	10 (19.23%)	9 (69.23%)	4 (15.38%)
	na-md-MCI		3 (5.45%)	3 (5.77%)	2 (15.38%)	1 (3.85%)
**Subjectively impaired domains**			1.47 (1.42)<br>[0 - 5]	1.88 (1.25)<br>[0 - 3]	2 (1.33)<br>[0 - 4]	1.52 (1.43)<br>[0 - 5]
**BDI-II**			6.28 (7.35)<br>[0 - 41]	6.55 (7.47)<br>[0 - 41]	9.75 (6.59)<br>[0 - 24]	6.96 (6.56)<br>[0 - 24]
**BAI**			4.66 (5.51)<br>[0 - 25]	4.81 (5.65)<br>[0 - 25]	6.73 (5.33)<br>[2 - 21]	4.63 (5.54)<br>[0 - 21]
**AES**			27.92 (8.50)<br>[17 - 50]	28.38 (8.55)<br>[17 - 50]	33.73 (9.61)<br>[18 - 50]	27.96 (8.95)<br>[17 - 50]
**pPD**	**probability**		0.96 (0.08)<br>[0.57 - 1]	0.98 (0.05)<br>[0.80 - 1]	0.98 (0.04)<br>[0.85 - 1]	0.97 (0.07)<br>[0.74 - 1]
**pDLB**	**essential**	1	11 (20%)	13 (25.00%)	13 (100%)	7 (26.92%)
	**core**	1	24 (43.64%)	22 (42.31%)	3 (23.08%)	4 (15.38%)
		2	31 (56.36%)	30 (57.69%)	10 (76.92%)	22 (84.62%)
	**biomarker**	1	41 (74.55%)	38 (73.08%)	10 (76.92%)	17 (65.38%)
		2	14 (25.45%)	14 (26.92%)	3 (23.08%)	9 (34.62%)
	**supportive**	1	7 (12.73%)	6 (11.54%)	3 (23.08%)	4 (15.38%)
		2	28 (50.91%)	28 (53.85%)	3 (23.08%)	10 (38.46%)
		3	13 (23.64%)	13 (25%)	6 (46.15%)	7 (26.92%)
		4	1 (1.82%)	1 (1.92%)	1 (7.69%)	1 (3.85%)
		5	1 (1.82%)	1 (1.92%)	0 (0%)	0 (0%)
**pMSA**	**essential**	1	55 (100%)	52 (100%)	13 (100%)	26 (100%)
	**non-motor**	1	32 (58.18%)	29 (55.77%)	8 (61.54%)	15 (57.69%)
		2	23 (41.82%)	23 (44.23%)	5 (38.46%)	11 (42.31%)
	**motor**	0	20 (36.36%)	18 (34.62%)	3 (23.08%)	0 (0%)
		1	35 (63.64%)	34 (65.38%)	10 (76.92%)	26 (100%)
	**exclusion criteria**	0	38 (69.81%)	35 (67.31%)	8 (61.54%)	26 (100%)
		1	17 (30.91%)	17 (32.69%)	5 (38.46%)	0 (0%)

Notes. Data are mean (standard deviation) [range] or n (%). Group-wise data is based on those fulfilling the prodromal disease definitions of pPD, pDLB, and pMSA, irrespective of overlapping diagnoses.

AES, Apathy Evaluation Scale; a-sd-MCI, amnestic single-domain mild cognitive impairment; a-md-MCI, amnestic multi-domain mild cognitive impairment; BAI, Beck Anxiety Inventory; BDI-II, Beck Depression Inventory; MoCA, Montréal Cognitive Assessment; na-md-MCI, non-amnestic multi-domain mild cognitive impairment; RBD, rapid eye movement behavior disorder; MDS-UPDRS-III, Movement Disorder Society Unified Parkinson's Disease Rating Scale Part 3; pDLB, prodromal dementia with Lewy bodies; pMSA, prodromal multiple system atrophy; pPD, prodromal Parkinson's disease.

SUPPLEMENTARY MATERIAL 4 (continued)

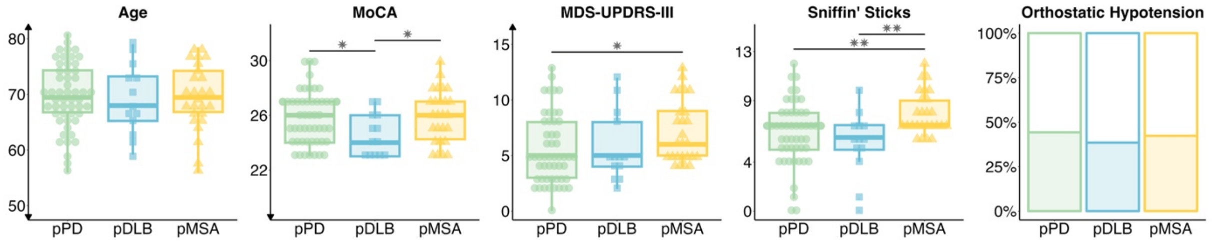

Sample characteristics irrespective of overlap between disease definitions. Age in years, MoCA (Montréal Cognitive Assessment) scores, MDS-UPDRS-III (Unified Parkinson's Disease Rating Scale Part 3) scores, Sniffin' Sticks score and presence of orthostatic hypotension in percent of individuals fulfilling the prodromal disease definitions of pPD (i.e., pPD only, green), pDLB (i.e., pDLB&pPD and pDLB&pPD&pMSA, blue), and pMSA (i.e., pMSA and pMSA&pPD, yellow). Dots/squares/triangles represent individual scores. The group-wise boxplots visualize the within-group median, the hinges represent the corresponding first and the third quartile, and the whiskers are 1.5 times the inter-quartile range. Asteriks represent significance of pairwise Mann-Whitney U tests, ^{+}p <.100, *p <.050, ** $p \leq .010$, *** $p \leq .001$

SUPPLEMENTARY MATERIAL 5

Sensitivity analyses excluding hyposmia rather than anosmia for prodromal multiple system atrophy

While the published pMSA criteria[27] consistently specify anosmia rather than hyposmia as the exclusion criterion, they lack a precise definition for how to operationalize anosmia in clinical assessments. Based on the terminology in Wenning, Stankovic [27], we added a sensitivity analysis applying the cut-off for hyposmia (<10) rather than for anosmia (<6) in our olfactory testing with the Sniffin' Sticks as an exclusion criterion for pMSA.

This significantly reduced the number of pMSA classifications. At least one of the prodromal disease definitions was fulfilled in 96.4% of the individuals with iRBD, and 94.5% could be classified as pPD. 'pPD only' was fulfilled in 63.6% of individuals, 'pMSA only' in 1.8%. In contrast, all individuals with pDLB fulfilled at least one additional prodromal definition. Overall, 30.9% fulfilled more than one definition: 7.3% were classified as pMSA&pPD, 21.8% as pDLB&pPD, and 1.8% fulfilled all three prodromal definitions (pPD&pDLB&pMSA). The overlap is visualized in the Figure below. Particularly group differences in cognitive [$\chi^2(2) = 9.06$, $p = .011$, (pPD = pMSA) > (pDLB = pMSA)] and olfactory testing [$\chi^2(2) = 14.00$, $p < .001$, (pPD = pDLB) < pMSA] persisted, while group differences in the MDS-UPDRS-III [$\chi^2(2) = 0.96$, $p = .619$, pPD = pDLB = pMSA] diminished. For details, please refer to the Table below.

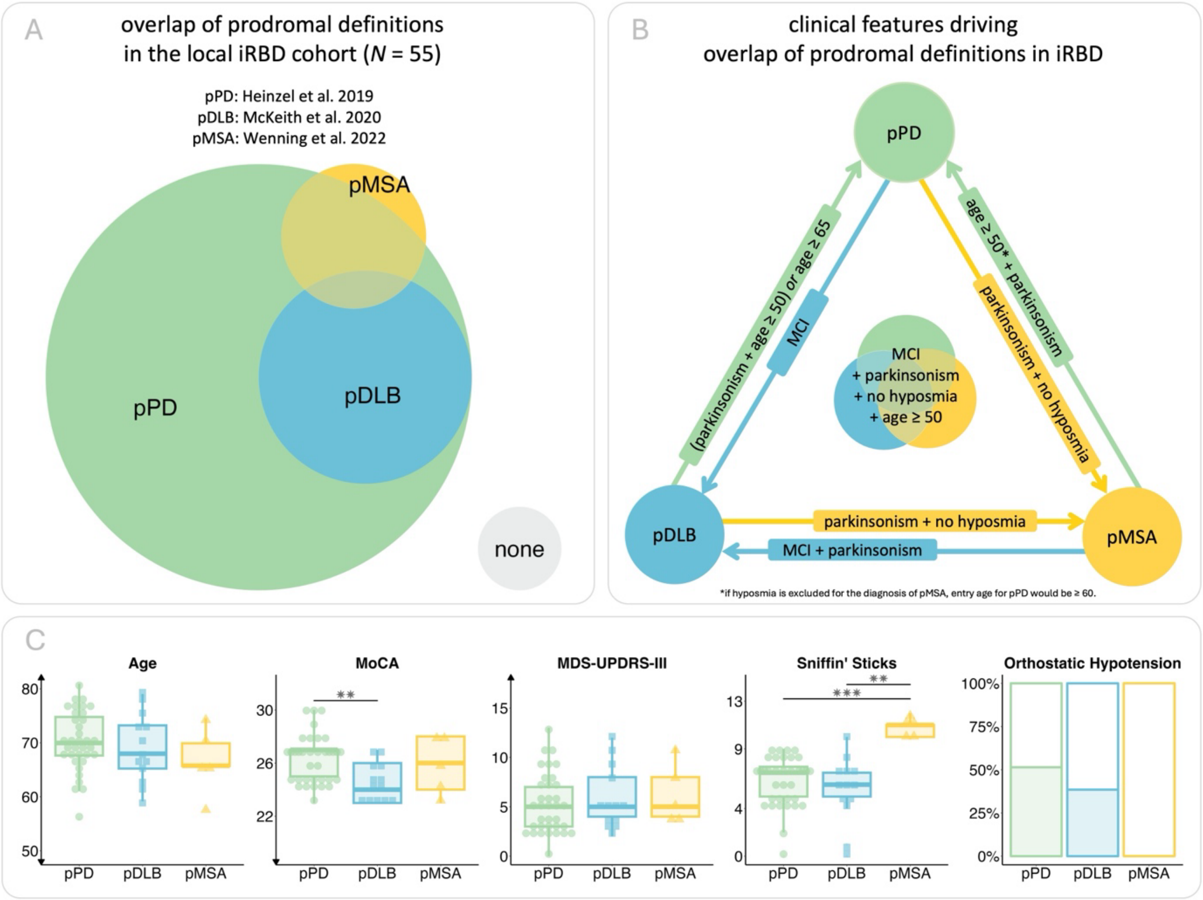

Sample characteristics following sensitivity analyses excluding hyposmia rather than anosmia for prodromal multiple system atrophy

			ALL<br>*N* = 55	pPD*<br>*n* = 35	pDLB*<br>*n* = 13	pMSA*<br>*n* = 5	*p*
**Age**			69.41 (5.75) [55.90 - 80.93]	70.20 (5.50) [55.90 - 80.93]	69.19 (6.27) [59.15 - 79.02]	66.70 (6.24) [57.63 - 74.53]	.374
**Sex**	female		7 (12.73%)	5 (14.29%)	2 (15.38%)	0 (0%)	.653
	male		48 (87.27%)	30 (85.71%)	11 (84.62%)	5 (100%)	
**Years of Education**			15.62 (3.17) [7 - 22]	16.23 (3.12) [10 - 22]	13.54 (2.91) [7 - 19]	15.80 (2.71) [12.00 - 19.50]	.045
**Time since first reported RBD symptoms in years**			8.02 (6.64) [1.43 – 28.94]	8.37 (7.17) [1.43 - 28.94]	6.20 (5.40) [1.77 - 20.13]	11.00 (5.13) [3.65 - 15.60]	.250
**Sniffin' Sticks**			6.48 (2.55) [0 - 12]	6.20 (2.07) [0 - 9]	5.54 (2.70) [0 - 10]	10.80 (0.84) [10 - 12]	<.001
**Olfactory impairment**	anosmia		17 (31.48%)	12 (34.29%)	5 (38.46%)	0 (0%)	<.001
	hyposmia		31 (57.41%)	23 (65.71%)	7 (53.85%)	0 (0%)	
**Orthostatic Hypotension**			23 (42.59%)	18 (51.43%)	5 (38.46%)	0 (0%)	.087
**MDS-UPDRS-III**			5.43 (3.08) [0 - 13]	5.23 (3.10) [0 - 13]	5.85 (3.16) [2 - 12]	6.40 (3.05) [4 - 11]	<.619
**MoCA**			26.05 (2.07) [23 - 30]	26.43 (1.84) [23 - 30]	24.54 (1.56) [23 - 27]	25.80 (2.28) [23 - 28]	.011
**Level-II cognitive impairment**	a-sd-MCI		2 (3.64%)	0 (0%)	2 (15.38%)	0 (0%)	<.001
	a-md-MCI		10 (18.18%)	1 (2.86%)	9 (69.23%)	0 (0%)	
	na-md-MCI		3 (5.45%)	1 (2.86%)	2 (15.38%)	0 (0%)	
**Subjectively impaired domains**			1.47 (1.42) [0 - 5]	1.20 (1.51) [0 - 5]	2 (1.33) [0 - 4]	1.60 (1.67) [0 - 4]	.354
**BDI-II**			6.28 (7.35) [0 - 41]	5.51 (7.63) [0 - 41]	9.75 (6.59) [0 - 24]	4.80 (7.05) [0 - 17]	.057
**BAI**			4.66 (5.51) [0 - 25]	4.45 (5.88) [0 - 25]	6.73 (5.33) [2 - 21]	2.00 (1.41) [0 - 3]	.046
**AES**			27.92 (8.50) [17 - 50]	27.32 (7.71) [17 - 44]	33.73 (9.61) [18 - 50]	20.25 (2.75) [17 - 23]	.023
**pPD**	**probability**		0.96 (0.08) [0.57 - 1]	0.98 (0.04) [0.84 - 1.00]	0.98 (0.04) [0.85 - 1]	0.86 (0.10) [0.74 - 0.96]	.007
**pDLB**	**essential**	1	13 (23.64%)	0 (0%)	13 (100%)	0 (0%)	<.001
	**core**	1	24 (43.64%)	17 (48.57%)	3 (23.08%)	2 (40%)	.280
		2	31 (56.36%)	18 (51.43%)	10 (76.92%)	3 (60%)	
	**biomarker**	1	41 (74.55%)	24 (68.57%)	10 (76.92%)	5 (100%)	.313
		2	14 (25.45%)	11 (31.43%)	3 (23.08%)	0 (0%)	
	**supportive**	1	7 (12.73%)	2 (5.71%)	3 (23.08%)	1 (20%)	<.001
		2	28 (50.91%)	25 (71.43%)	3 (23.08%)	0 (0%)	
		3	13 (23.64%)	7 (20%)	6 (46.15%)	0 (0%)	
		4	1 (1.82%)	0 (0%)	1 (7.69%)	0 (0%)	
		5	1 (1.82%)	1 (2.86%)	0 (0%)	0 (0%)	
**pMSA**	**essential**	1	55 (100%)	35 (100%)	13 (100%)	5 (100%)	n.a.
	**non-motor**	1	32 (58.18%)	17 (48.57%)	8 (61.54%)	5 (100%)	.087
		2	23 (41.82%)	18 (51.43%)	5 (38.46%)	0 (0%)	
	**motor**	0	20 (36.36%)	15 (42.863%)	3 (23.08%)	0 (0%)	.106
		1	35 (63.64%)	20 (57.14%)	10 (76.92%)	5 (100%)	
	**exclusion criteria**	0	38 (69.81%)	0 (0%)	8 (61.54%)	5 (100%)	<.001
		1	17 (30.91%)	35 (100%)	5 (38.46%)	0 (0%)	

Notes. Data are mean (standard deviation) [range] or n (%). *P*-values of Kruskal-Wallis tests or Chi-squared tests are reported as appropriate. AES, Apathy Evaluation Scale; a-sd-MCI, amnestic single-domain mild cognitive impairment; a-md-MCI, amnestic multi-domain mild cognitive impairment; BAI, Beck Anxiety Inventory; BDI-II, Beck Depression Inventory; MoCA, Montréal Cognitive Assessment; na-md-MCI, non-amnestic multi-domain mild cognitive impairment; RBD, rapid eye movement behavior disorder; MDS-UPDRS-III, Movement Disorder Society Unified Parkinson's Disease Rating Scale Part 3; pDLB, prodromal dementia with Lewy bodies; pMSA, prodromal multiple system atrophy; pPD, prodromal Parkinson's disease.

* Groups were created so that each individual is represented only once. The pPD group includes those fulfilling the pPD criteria only, the pDLB group includes those fulfilling pDLB&pPD and pDLB&pPD&pMSA, and the pMSA group pMSA and pMSA&pPD. Two individuals did not fulfill any of the three prodromal disease definitions.

References

1. Goetz CG, Tilley BC, Shaftman SR, et al. Movement Disorder Society-sponsored revision of the Unified Parkinson's Disease Rating Scale (MDS-UPDRS): scale presentation and clinimetric testing results. Mov Disord 2008;23(15):2129-2170.
2. Wenning GK, Tison F, Seppi K, et al. Development and validation of the unified multiple system atrophy rating scale (UMSARS). Mov Disord 2004;19(12):1391-1402.
3. Society CCotAA, Neurology tAAo. Consensus statement on the definition of orthostatic hypotension, pure autonomic failure, and multiple system atrophy. Neurology 1996;46(5):1470-1470.
4. Visser M, Marinus J, Stiggelbout AM, Van Hilten JJ. Assessment of autonomic dysfunction in Parkinson's disease: the SCOPA-AUT. Mov Disord 2004;19(11):1306-1312.
5. Johns MW. A new method for measuring daytime sleepiness: the Epworth sleepiness scale. Sleep 1991;14(6):540-545.
6. Beck AT, Steer RA, Brown GK. Beck depression inventory-II. San Antonio 1996;78(2):490-498.
7. Beck AT, Steer R. Beck anxiety inventory (BAI). Überblick über Reliabilitäts-und Validitätsbefunde von klinischen und außerklinischen Selbst-und Fremdbeurteilungsverfahren 1988;7.
8. Marin RS, Biedrzycki RC, Firinciogullari S. Reliability and validity of the Apathy Evaluation Scale. Psychiatry research 1991;38(2):143-162.
9. Ismail Z, Agüera-Ortiz L, Brodaty H, et al. The Mild Behavioral Impairment Checklist (MBI-C): a rating scale for neuropsychiatric symptoms in pre-dementia populations. Journal of Alzheimer's disease: JAD 2017;56(3):929.
10. Kalbe E, Bintener C, Ophey A, et al. Computerized cognitive training in healthy older adults: Baseline cognitive level and subjective cognitive concerns predict training outcome. Health 2018;10(1):20-55.
11. Nasreddine ZS, Phillips NA, Bédirian V, et al. The Montreal Cognitive Assessment, MoCA: A brief screening tool for mild cognitive impairment. Journal of the American Geriatrics Society 2005;53(4):695-699.
12. Litvan I, Goldman JG, Tröster AI, et al. Diagnostic criteria for mild cognitive impairment in Parkinson's Disease: Movement Disorder Society Task Force guidelines. Mov Disord 2012;27(3):349-356.
13. McKeith IG, Ferman TJ, Thomas AJ, et al. Research criteria for the diagnosis of prodromal dementia with Lewy bodies. Neurology 2020;94(17):743-755.
14. Aschenbrenner S, Tucha O, Lange K. Regensburger Wortflüssigkeitstest Hogrefe Göttingen. Göttingen, Germany: Hogrefe; 2000.
15. Reitan R. Trail Making Test: Manual for administration and scoring. . Tucson, Arizona: Reitan Neuropsychology Laboratory, 1992.
16. Aebi C. Validierung der neuropsychologischen Testbatterie CERAD-NP: eine Multi-Center Studie [Dissertation]. Basel: University of Basel; 2002.
17. Rey A. L'examen psychologique dans les cas d'encéphalopathie traumatique.(Les problems.). Archives de psychologie 1941.
18. Strauss E, Sherman EM, Spreen O. A compendium of neuropsychological tests: Administration, norms, and commentary: American chemical society, 2006.
19. Sturm W, Willmes K, Horn W. Leistungsprüfsystem für 50–90jährige. Handanweisung. Göttingen: Hogrefe, 1993.
20. Wechsler D. WMS-R: Wechsler memory scale-revised: Manual: Psychological Corporation, 1984.
21. Helmstaedter C, Durwen H. VLMT: Verbaler Lern-und Merkfähigkeitstest: Ein praktikables und differenziertes Instrumentarium zur Prüfung der verbalen Gedächtnisleistungen. Schweizer Archiv für Neurologie, Neurochirurgie und Psychiatrie 1990.
22. Kalbe E, Reinhold N, Brand M, Kessler J. Aphasie-Check-Liste (ACL): Protokollheft, Testheft, Lösungsfolien, Vorlagen, Manual. Köln: ProLog, Therapie-und Lernmittel, 2002.
23. von Aster M, Neubauer A. Wechsler-intelligenztest für erwachsene: WIE; manual; übersetzung und adaptation der WAIS-III von David Wechsler: Pearson Assessment & Information, 2009.
24. Berg D, Postuma RB, Adler CH, et al. MDS research criteria for prodromal Parkinson's Disease. Mov Disord 2015;30(12):1600-1611.
25. Heinzel S, Berg D, Gasser T, et al. Update of the MDS research criteria for prodromal Parkinson's Disease. Mov Disord 2019;34(10):1464-1470.
26. Sateia MJ. International classification of sleep disorders. Chest 2014;146(5):1387-1394.
27. Wenning GK, Stankovic I, Vignatelli L, et al. The movement disorder society criteria for the diagnosis of multiple system atrophy. Mov Disord 2022;37(6):1131-1148.
